# SOCE induced calcium overload regulates autophagy in acute pancreatitis via calcineurin activation

**DOI:** 10.1038/s41419-017-0073-9

**Published:** 2018-01-19

**Authors:** Zhen-Dong Zhu, Tao Yu, Hua-Jing Liu, Jing Jin, Jun He

**Affiliations:** 1 Department of Histology and Embryology, School of Basic Medicine, Tongji Medical College, Huazhong University of Science and Technology, Wuhan, China; 2 Wuhan Children’s Hospital (Wuhan Maternal and Child Healthcare Hospital), Tongji Medical College, Huazhong University of Science and Technology, Wuhan, China

## Abstract

Acute pancreatitis (AP) is an acute inflammatory process of the pancreas that is characterized by inflammation, edema, vacuolization and necrosis, which has significant morbidity and lethality. The pathogenesis of AP has not been established completely. An early and critical feature of AP is the aberrant signaling of Calcium (Ca^2+^) within the pancreatic acinar cell, termed Ca^2+^ overload. Store-operated Ca^2+^ (SOC) channels are the principal Ca^2+^ influx channels that contribute to Ca^2+^ overload in pancreatic acinar cells. Store-operated Ca^2+^ entry (SOCE) has been proved to be a key pathogenic step in AP development that leads to trypsin activation, inflammation and vacuolization. However, the molecular mechanisms are still poorly understood. By establishing Ca^2+^ overload model and mouse AP model using caerulein, we found that caerulein triggered SOCE via inducing interaction between STIM1 and Orai1, which activated calcineurin (CaN); CaN activated the nuclear factor of activated T cells (NFAT) and transcription factor EB (TFEB), thus promoting the transcriptional activation of multiple chemokines genes and autophagy-associated genes respectively. To the best of our knowledge, this is the first evidence showing that SOCE activates TFEB via CaN activation, which may have noticeable longer-term effects on autophagy and vacuolization in AP development. Our findings reveal the role for SOCE/CaN in AP development and provide potential targets for AP treatment.

## Introduction

Acute pancreatitis (AP) is an acute autodigestive disease caused by multiple factors, which is generally accompanied by inflammation, edema, hemorrhage and even necrosis in its own tissue or remote organs^1–4^. The mechanism which initiates the AP process remains to be established. In the initiation of AP, Ca^2+^ influx is markedly increased under stimuli of cholecystokinin (CCK), bile acid, alcohol metabolites or some other causes^5–7^. Ca^2+^ is a versatile carrier of signals regulating many aspects of cellular activity and contributing in controlling cellular functions including gene expression, enzymes activity and exocrine functions in pancreatic acinar cells^8–10^. Ca^2+^ overload, caused by intracellular Ca^2+^ homeostasis disruption, is considered to be a key contributor to pancreatic acinar cell injury due to prolonged and global intracellular Ca^2+^ concentration ([Ca^2+^]_i_) elevation that leads to trypsin activation, inflammation, necrosis and vacuolization^7,11–13^.

In both excitable and especially in non-excitable cells, store-operated calcium entry (SOCE) is a ubiquitous Ca^2+^ entry pathway that is activated in response to depletion of endoplasmic reticulum (ER) Ca^2+^ stores, and is essential for the regulation of cell growth and proliferation, exocytosis, modulation of enzymatic activity and motility, and the immune response^14,15^. Previous studies have demonstrated a role for store-operated Ca^2+^ (SOCs) channels as the principal Ca^2+^ influx channel in pancreatic acinar cells^6,7^. The core components have been debated since the discovery of SOC channel. Early studies suggested that transient receptor potential canonical (TRPC) channel is the dominant channel in SOCE, which is the primary contributor of Ca^2+^ overload^15–17^. However, recent studies indicate Orai1 is the principal SOCE channel in the pancreatic acinar cell, the opening of which is coordinated by stromal interaction molecule-1 (STIM1), after emptying of the ER Ca^2+^ store^11,18–20^. Inhibition of Orai1 channels prevents cytosolic calcium overload-associated injury of pancreatic acinar cells and acute pancreatitis^11,19,21^. However, the downstream mechanisms of calcium release-activated calcium channel (CRAC) in AP is still not fully understood.

Calcineurin (CaN) is a widely distributed Ca^2+^/calmodulin (CaM)-dependent protein phosphatase that can be activated by the [Ca^2+^]_i_ elevation and regulates multiple physiological and pathological processes^22–24^. In the exocrine pancreas, the Ca^2+^–CaM–CaN pathway is required for enzyme secretion and cell growth^25,26^. Nuclear factor of activated T cells (NFAT) and transcription factor EB (TFEB) are the target proteins of CaN, the activation of which regulates inflammation and autophagy, respectively^27,28^. CaN activation dephosphorylated NFAT, leading to translocation of NFAT from cytoplasm to nucleus, transcriptional activation of chemokine-related genes and inflammatory infiltration^27^.

Vacuolization is one of the typical pathological features of AP and previous studies showed that the majority of vacuoles were autophagic in origin^29–32^. TFEB is an autophagy-related transcription factor whose overexpression induced the transcription of autophagy-related genes and autophagy activation^33^. Autophagy is a lysosome-driven, multistep and adaptive process whereby the bulk cytoplasmic contents including intracellular membrane structures, protein aggregates and damaged organelles are degraded to maintain cellular homeostasis^34,35^. The normal level of autophagy can protect cells from environmental stimuli, but continued excessive or insufficient autophagy could lead to disease^35,36^. Previous studies have revealed that autophagy is induced but impaired in AP because of its inefficient flux resulting from defective function of lysosomes^31,32,37^. Impaired autophagy is involved in the process of trypsinogen activation^30,32^ and inflammation^38,39^ during early stages of pancreatitis, and results in the accumulation of large vacuoles in acinar cells^32,37^, suggesting that autophagy plays a crucial role in the pathogenesis of pancreatitis.

Caerulein, a CCK receptor agonist, is widely used in experimental AP. In Ca^2+^ overload cellular model and mouse AP model induced by caerulein, we found that caerulein triggered SOCE via inducing interaction between STIM1 and Orai1, which activated CaN; CaN then induced the dephosphorylation of TFEB /NFAT and nuclear translocation, which in turn promoted the transcriptional activation of multiple chemokines genes and autophagy associated genes. These findings suggest that TFEB may play a key role in the longer term effects of SOCE / CaN on autophagy and vacuolisation in AP development.

## Results

### Caerulein triggers SOCE via inducing the interaction of STIM1 with Orai1

**Fig. 1:**
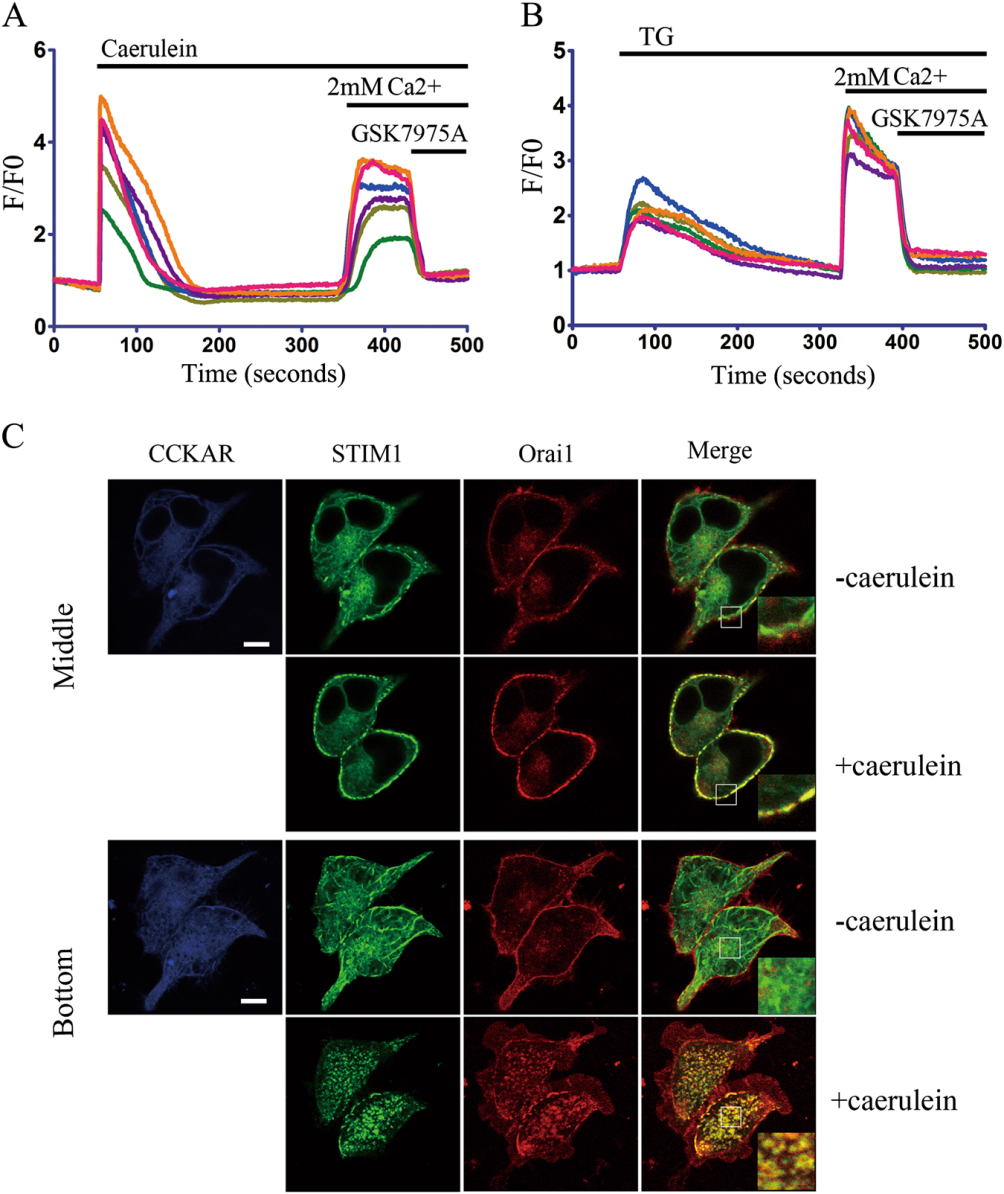
Caerulein triggers SOCE via inducing interaction between STIM1 and Orai1 **a**, **b** Effects of incubation with 10 μM GSK7975A under stimulation with 10 nM **a** caerulein or **b** 2 μM TG in 293T cells transfected with CCKAR-mCherry. Where indicated, Ca^2+^ was added to a final concentration of 2 mM. **c** Representative confocal images of HEK293T cells co-expressed with CCKAR-CFP, STIM1-YFP and Orai1-mCherry after stimulating with 10 nM caerulein. Scale bar, 10 μm

Orai1 mediates the caerulein-induced SOCE in pancreatic acinar cells and results in pancreatic injury^21^. Pharmacological and functional studies demonstrate that cholecystokinin A receptor (CCKAR) is the dominant type of CCK receptor located in the membrane of pancreatic acinar cells^40^. To gain visualized evidence that caerulein can trigger SOCE by promoting the interaction between STIM1 and Orai1, the plasmids of CCKAR were constructed in the present study, whose function was validated by confocal calcium imaging (Fig. 1a, b), and then the plasmids CCKAR-CFP, STIM1-YFP and Orai1-mCherry were co-overexpressed in 293T cells. In physiological status, STIM1 showed tubular-like structures in cytoplasm and Orai1 uniformly distributed at plasma membrane, and there were no co-localization between these two proteins (Fig. 1c). At 4 min after stimulating with 10 nM caerulein, STIM1 formed puncta-like co-localization with Orai1 (Fig. 1c). These data suggest that caerulein promoted the interaction between STIM1 and Orai1.

### CaN activation depends on caerulein-induced SOCE

**Fig. 2:**
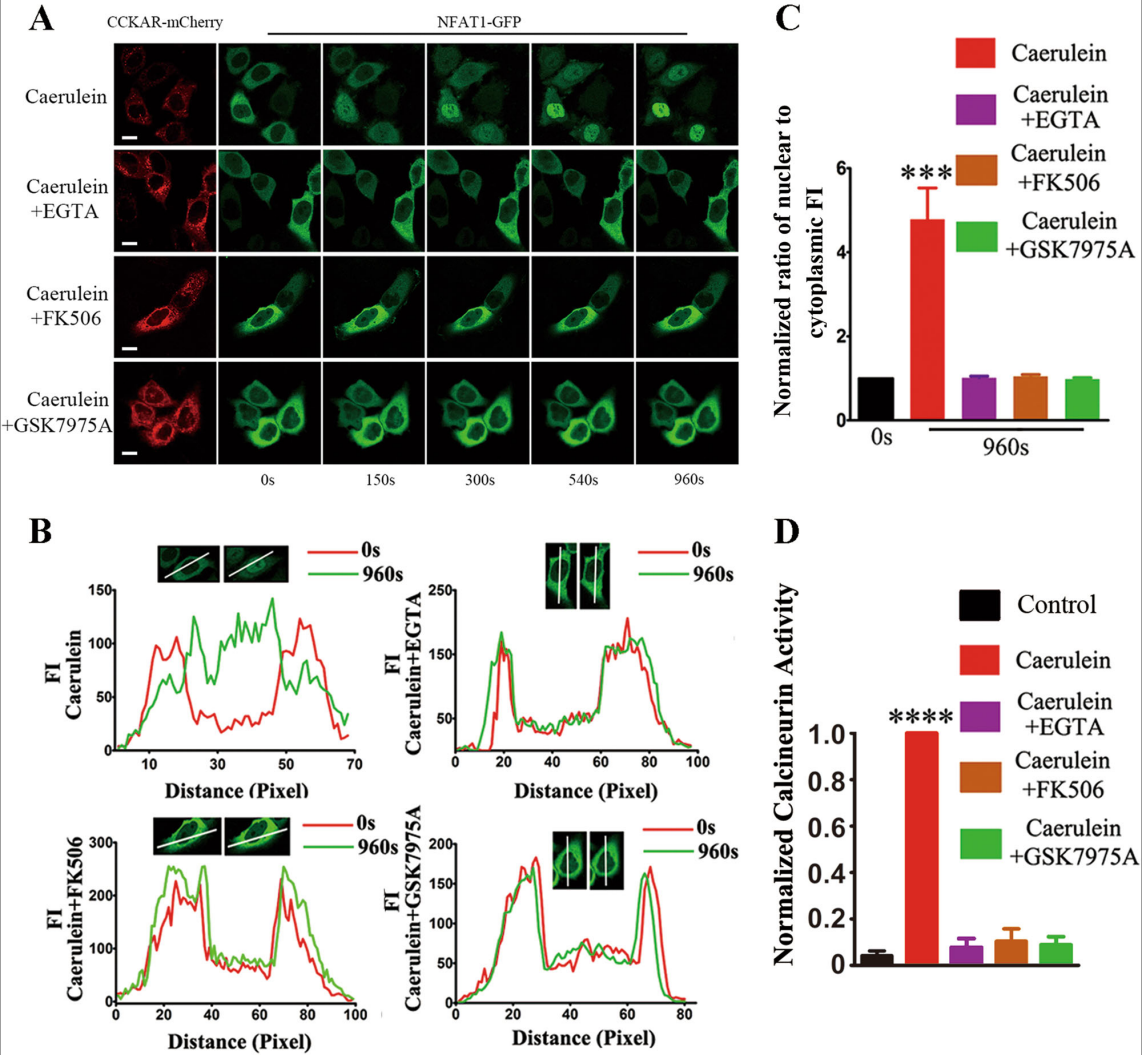
CaN activation depends on caerulein-induced SOCE **a** Confocal live cell imaging shows dynamic subcellular distribution of NFAT in the different groups of Hela cells co-expressing CCKAR-mCherry and NFAT1-GFP: four groups were pretreated with DMSO, EGTA (5 mM), FK506 (10 μM) and GSK7975A (10 μM) respectively for 30 min; images were captured every 30 s within 20 min; the dotted boxes show the cells measured by line profile. Scale bar, 10 μm. **b** Spatial profiles of fluorescence intensity (FI) of NFAT-GFP along the lines imposed on the images. **c** The graph shows the normalized ratio of average FI of nuclei to cytoplasm (N/C) in the different groups of cells: DMSO group (*n* = 9), EGTA group (*n* = 10), FK506 group (*n* = 11) and GSK7975A group (*n* = 10). The data were from three independent experiments and expressed as mean ± SEM. ***P < 0.001 vs 0 s, caerulein plus EGTA, caerulein plus FK506, or caerulein plus GSK7975A. **d** The CaN activity in different groups of pancreatic acinar cells detected by using the activity assay kit. The data were from three independent experiments and expressed as mean ± SEM. ****P < 0.0001 vs control, caerulein plus EGTA, caerulein plus FK506, or caerulein plus GSK7975A

CaN is a Ca^2+^/CaM-dependent protein phosphatase that can be activated by the [Ca^2+^]_i_ elevation^23^. Significantly, CaN inhibition by FK506 can reduce the severity of inflammation, edema and vacuolation in caerulein-induced AP, suggesting that CaN may be a key regulator in SOCE-associated pancreatic injury^41,42^. NFAT are transcription factors of chemokines, whose dephosphorylation by CaN results in nuclear translocation and transcriptional activation^43^. To explore whether SOCE is necessary for CaN activation, CCKAR-mCherry and NFAT1-GFP were co-overexpressed in Hela cells, and confocal live cell imaging was used to monitor the dynamic subcellular localization of NFAT. After treatment with caerulein, we observed that NFAT1 obviously translocated from cytoplasm to nucleus within 150 s, and 540 s later, most of NFAT1 translocated to nucleus, which was completely blocked in the presence of FK506 (CaN inhibitor) or EGTA or GSK7975A (Fig. 2a). Line profiles of fluorescence intensity indicated that the NFAT in nucleus was significantly increased after stimulating with caerulein; meanwhile, negligible changes were found in the cells pretreated with EGTA or FK506 or GSK7975A (Fig. 2b) and the quantitative data showed consistency with line profiles of fluorescence intensity (Fig. 2c). Calcineurin activity of pancreatic acinar cells was also analyzed by the use of caerulein with/without FK506 or EGTA or GSK7975A (Fig. 2d). Our results further supported that caerulein-induced CaN activation depends on extracellular Ca^2+^ entry, in which SOCE played a decisive role.

### TFEB may be a contributor to the increase of autophagosome in AP

**Fig. 3:**
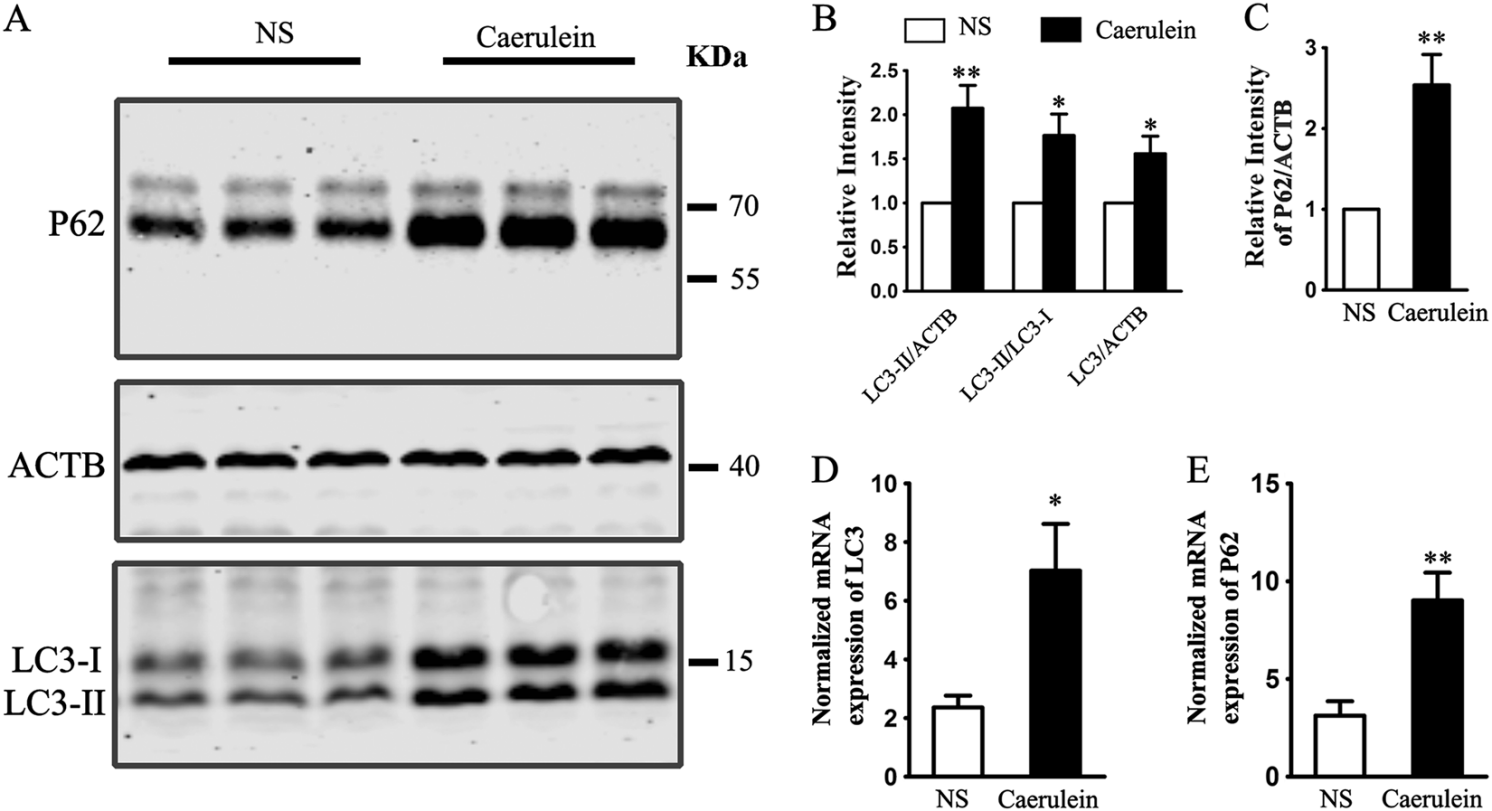
**The protein and mRNA levels of LC3 and P62 were up-regulated in AP. a** Expression of LC3 and P62 in pancreatic tissues. **b**, **c** Quantitative analyses showed that AP increased the total level of LC3, LC3II and SQSTM1/P62; *n* = 6. **d**, **e** Real-time fluorescent quantitative PCR showed the mRNA level of LC3 and SQSTM1/P62; *n* = 5. The data were expressed as mean ± SEM, *p < 0.05, **p < 0.01 vs NS

The majority of vacuoles were autophagic in origin^30^. LC3II, an autophagosome membrane-bound protein, is considered the most reliable autophagosome-associated protein marker, while SQSTM1/P62 serves as an index of autophagic degradation^44^. The levels of LC3II and SQSTM1/P62 in the present study were detected by western blotting. We observed that the levels of LC3II and SQSTM1/P62 were significantly increased in AP (Fig. 3a–c), which suggests that the autophagosome was increased but autophagy influx impaired. We further measured the mRNA level of LC3II and SQSTM1/P62. Data of real-time PCR showed that the mRNA of LC3II and SQSTM1/P62 were increased in AP (Fig. 3d, e), which implies that supramaximal caerulein might increase the autophagosomes both by increasing the expression of autophagy-related genes and inducing inefficient degradation.

**Fig. 4:**
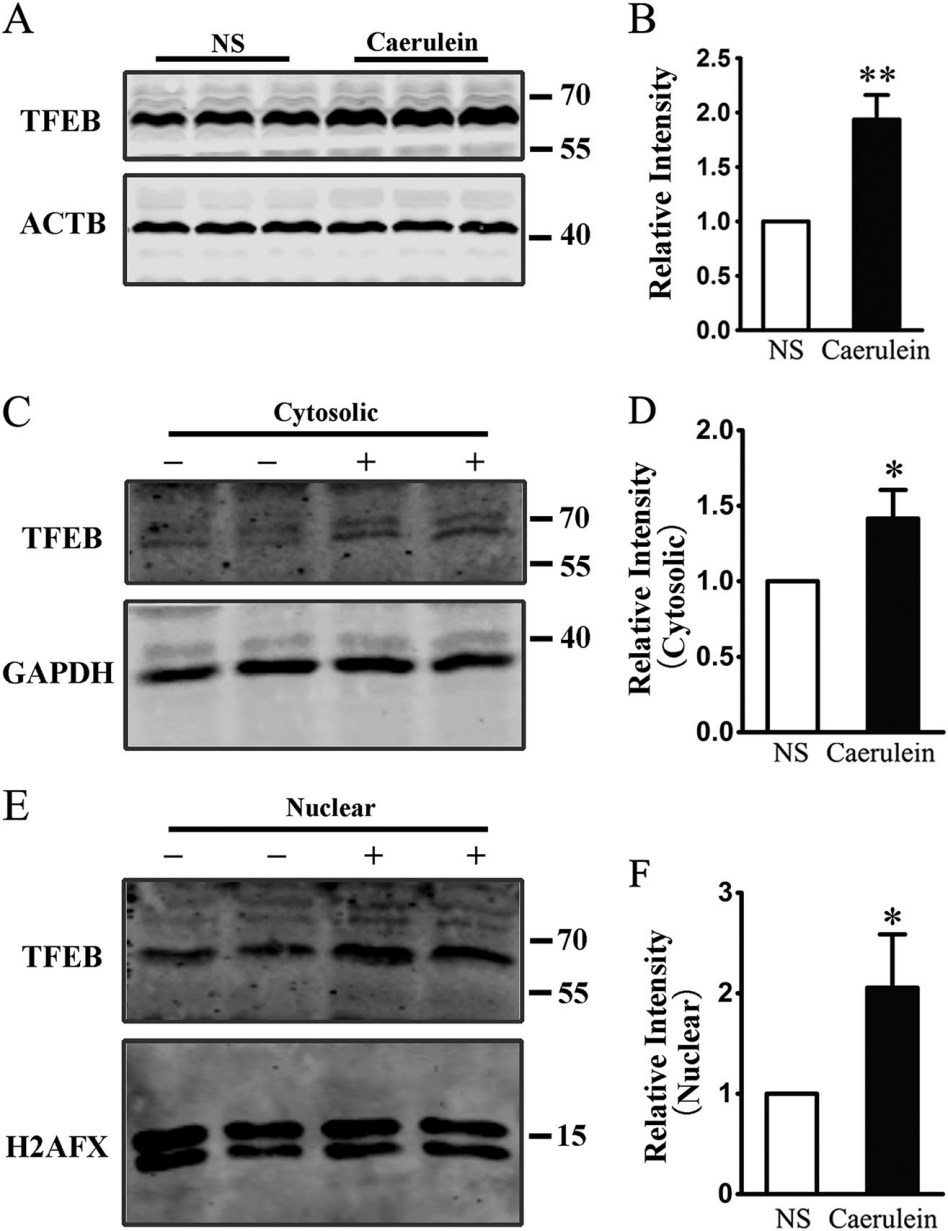
**Caerulein increased expression of TFEB both in cytoplasm and nucleus. a**, **b** Expression of TFEB in pancreatic tissues; *n* = 6. **c**, **d** Expression of cytosolic TFEB in pancreatic acinar cells stimulating for 6 h with 10 nM caerulein or NS; *n* = 3. **e**, **f** Expression of nuclear TFEB in pancreatic acinar cells stimulating for 6 h with 10 nM caerulein or NS; *n* = 3. *P < 0.05, **p < 0.01 vs NS

TFEB is an autophagy-related transcription factor, whose overexpression induced the transcription of autophagy-related genes and autophagy activation^33^. To explore the reason that caerulein increased the expression of autophagy-related genes, we measured the level of TFEB in pancreas by western blotting (Fig. 4a), and found that the level of TFEB was significantly increased in the pancreases of pancreatitic mice (Fig. 4b). TFEB dephosphorylation leads to a translocation from cytoplasm into the nucleus, which activates its target genes^45^. To further verify whether TFEB was led to nuclear tranlocation in caerulein-AP, we measured the level of cytosolic and nulear TFEB in pancreatic acinar cells. Unexpectedly, caerulein was found to increase both the levels of nuclear and cytosolic TFEB (Fig. 4c–f), which means some other methods should be utilized to determine whether caerulein can induce TFEB nuclear tranlocation.

### SOCE-induced CaN activation regulates nuclear translocation of TFEB

**Fig. 5:**
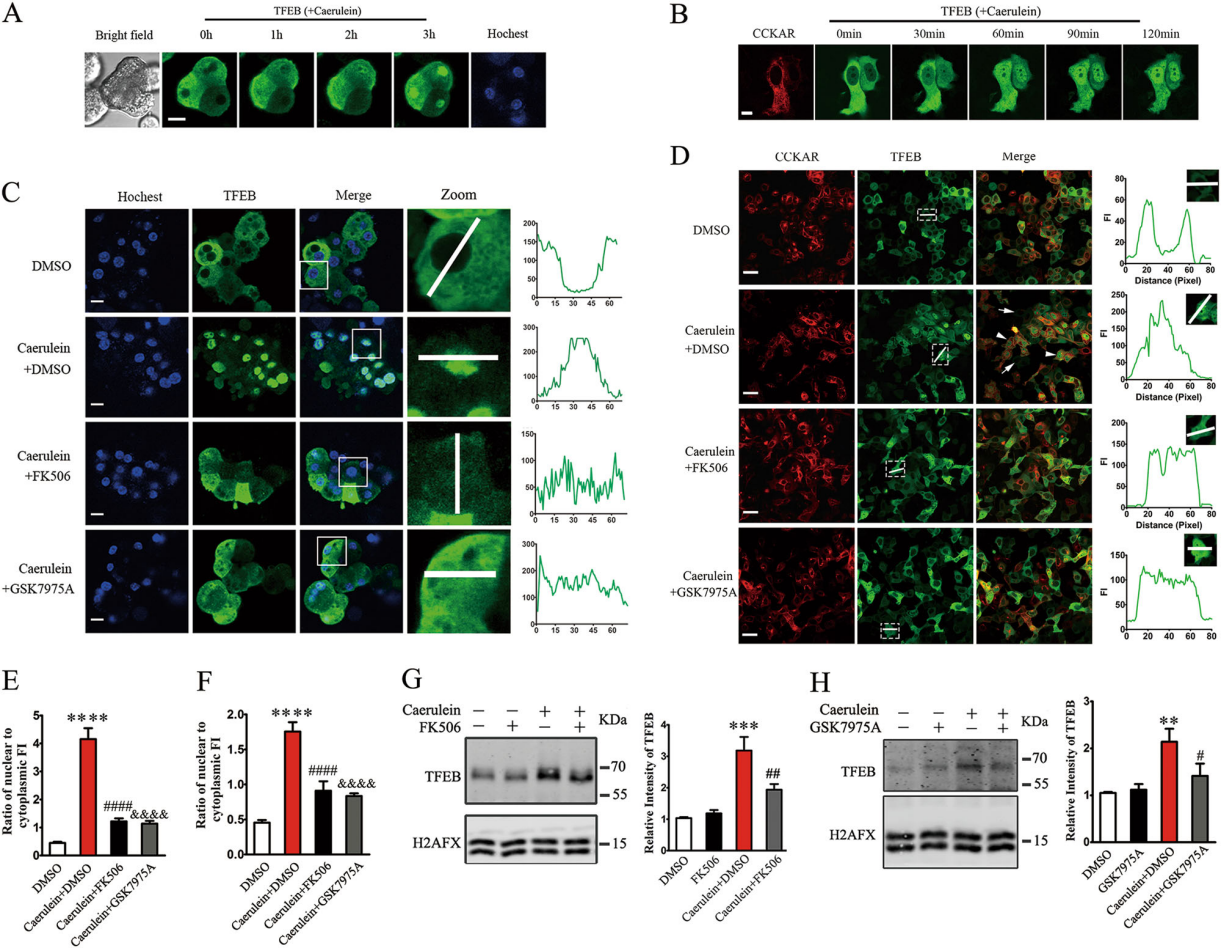
**SOCE regulates TFEB nuclear translocation via CaN activation.** Pancreatic acinar cells were tranfected with AAV-TFEB-EGFP for 24 h; Hela cells were co-transfected with CCKAR-mCherry and TFEB-GFP for 48 h. **a** Time-lapse confocal microscopy imaging shows the dynamic subcellular distribution of TEFB in pancreatic acinar cells after stimulating with 10 nM caerulein with a 1 h time interval between two contiguous images; Scale bar, 10 μm. **b** Time-lapse confocal microscopy imaging shows the dynamic subcellular distribution of TEFB in Hela cells after stimulating with 10 nM caerulein with a 30 min time interval between two contiguous images; Scale bar, 10 μm. **c** Pancreatic acinar cells pretreated with DMSO or 10 μM FK506 or 10 μM GSK7975A for 30 min were stimulated for 3 h with 10 nM caerulein; confocal microscopy imaging shows the effects of FK506 or GSK7975A in caerulein-induced TFEB nuclear translocation; “zoom” panel shows the cells in the white boxes. The right panel shows spatial profiles of fluorescence intensity (FI) of TFEB along the lines imposed on the images of “zoom in”. Scale bar, 10 μm. **d** Hela cells pretreated with DMSO or 10 μM FK506 or 10 μM GSK7975A for 30 min were stimulated for 3 h with 10 nM caerulein; confocal microscopy imaging shows the effects of FK506 or GSK7975A in caerulein-induced TFEB nuclear translocation. The white arrows show the subcellular distribution of TFEB in the cells expressing TFEB-GFP only and the arrowheads show the cells co-expressing CCKAR-mCherry and TFEB-GFP. The right panel shows spatial profiles of FI of TFEB-GFP along the lines imposed on the images (the dotted boxes). Scale bar, 40 μm. **e** The graph shows the ratio of average FI of nuclei to cytoplasm (N/C) of pancreatic acinar cells from DMSO group (*n* = 19), caerulein plus DMSO group (*n* = 22), caerulein plus FK506 group (*n* = 17) and caerulein plus GSK7975A group (*n* = 19). **** *P* < 0.0001 vs control; ####*p* < 0.0001, &&&& *p* < 0.0001 vs caerulein group. **f** The graph shows the ratio of average FI of nuclei to cytoplasm (N/C) of Hela cells from DMSO group (*n* = 30), caerulein plus DMSO group (*n* = 23), caerulein plus FK506 group (*n* = 27) and caerulein plus GSK7975A group (*n* = 30). ****P < 0.0001 vs control; #### *p* < 0.0001, &&&& *p* < 0.0001 vs caerulein group. **g**,** h** Pancreatic acinar cells pretreated with 10 μM FK506 or 10 μM GSK7975A or DMSO for 30 min were stimulated for 3 h with 10 nM caerulein. Western blotting shows the expression of TFEB in the nuclear of pancreatic acinar cells and quantitative analyses show that FK506/GSK7975A decreased the level of TFEB in nuclei of pancreatic acinar cells. The data were from at least 3 independent experiments and expressed as mean ± SEM. ***P < 0.001, **p < 0.01 vs cells treated with DMSO group. ##*P* < 0.01, #*p* < 0.05 vs caerulein group

To explore the effects of caerulein on TFEB nuclear translocation, TFEB-GFP and CCKAR-mCherry were co-overexpressed in Hela cells, and AAV-TFEB-EGFP was used to transfect pancreatic acinar cells. By confocal live cell imaging, we monitored the dynamic subcellular localization of TFEB and found that TFEB gradually translocated to the nucleus after stimulating with caerulein (Fig. 5a, b). Most of TFEB translocated into nuclei 3 h after stimulating with caerulein, whereas there was no obvious nuclear translocation in Hela cells that singly expressed TFEB, which further confirmed that TFEB translocation depends on caerulein stimulation (Fig. 5d). TFEB, which was activated by CaN-mediated dephosphorylation, translocated to the nucleus and up-regulated the expression of autophagy-related genes^28^. To study the correlation of caerulein-induced TFEB nuclear translocation with CaN, we used FK506 to inhibit the activation of CaN or GSK7975A to inhibit SOCE. It was found that inhibition of either CaN or SOCE decreased the nuclear translocation of TFEB (Fig. 5c–f). Furthermore, by using western blotting, endogenous TFEB was measured in pancreatic acinar cells. We found that inhibition of CaN or SOCE partially restored the level of nuclear TFEB (Fig. 5g, h). These data strongly suggest that caerulein induces TFEB nuclear translocation via SOCE-activated CaN activation.

### CaN inhibition reduces the level of LC3II

**Fig. 6:**
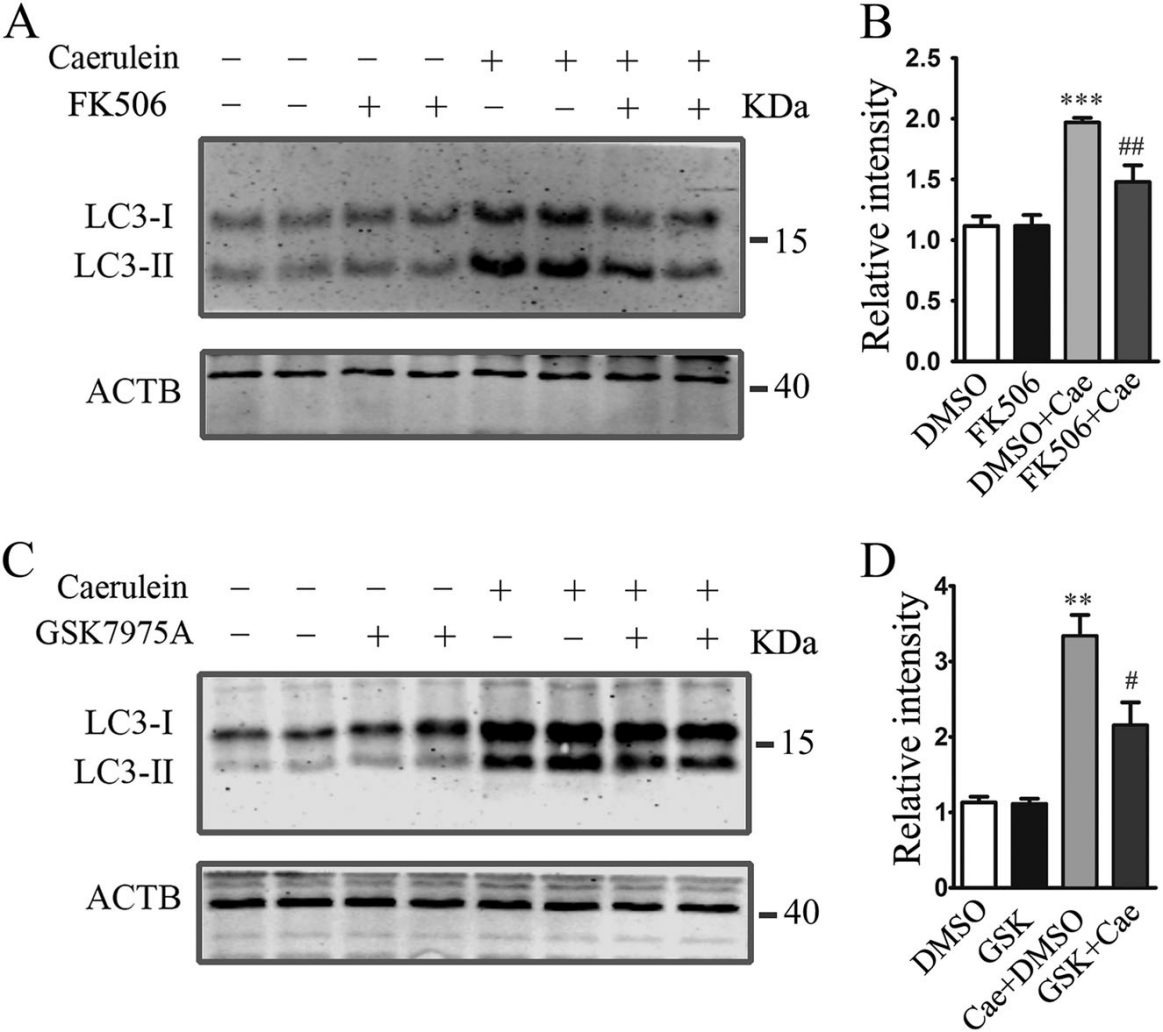
**CaN inhibition reduced the formation of autophagosome.** Pancreatic acinar cells were pretreated with 10 μM FK506 or 10 μM GSK7975A or DMSO for 30 min before stimulating with 10 nM caerulein, and 6 h later, the cell lysates were prepared for western blotting. **a** Expression of LC3II in FK506 and/or caerulein-treated pancreatic acinar cells. **b** Quantitative analyses showed that FK506 decreased the level of LC3II in pancreatic acinar cells. **c** Expression of LC3II in GSK7975A and/or caerulein-treated pancreatic acinar cells. **d** Quantitative analyses showed that GSK7975A decreased the level of LC3II in pancreatic acinar cells. The data were from three independent experiments and expressed as mean ± SEM. **P < 0.01, ***p < 0.001, vs DMSO group; ^##^*p* < 0.01, ^#^*p* < 0.05, vs caerulein group

To observe the effects of CaN activation in the formation of autophagosome in AP, pancreatic acinar cells were acutely dissociated and pretreated with FK506 before stimulating with caerulein, and western blotting was used to detect the protein level of LC3II. As shown in Fig. 6a, b, FK506 attenuated the increase of LC3II induced by caerulein. As CaN activation was regulated by STIM1–Orai1-induced SOCE, GSK7975A was used to inhibit Orai1 in pancreatic acinar cells, and western blotting results showed that GSK7975A also attenuated the caerulein-induced increase of LC3II (Fig. 6c, d).

## Discussion

SOC is the primary Ca^2+^ influx channel in non-excitable cells^14,15^, which induced sustaining Ca^2+^ elevation and triggered Ca^2+^ overload under pathological stimulus^7,11,12^. Since the discovery of SOCE, the components and the mechanism underlying the regulation of SOCs has been intensely debated. One form of SOCs is the TRPC channel, which is a kind of non-selective cation channel^46,47^. Previous studies showed that pancreatic acinar cells endogenously express TRPC1, TRPC3 and TRPC6^48^, and inhibition of TRPC3 protected pancreatic acinar cells from Ca^2+^-dependent toxicity^16,17^. The best-characterized SOCs is the CRAC, which is a highly selective Ca^2+^ channel^14,15^. Recently, two molecular components of CRAC have been found to mediate SOCE after Ca^2+^ store depletion: STIM that is located in the membrane of the ER as the Ca^2+^ sensor in the ER, along with Orai proteins that are located in the plasma membrane as the pore-forming subunits of CRAC channel^14^. STIM–Orai1 interaction in pancreatic acinar cells was first, and directly, demonstrated by Lur et al.^18^ and data of recent studies indicate that the dominant store-operated current is of the Ca^2+^-selective CRAC channel type and Orai1 is the principal SOCE channel in the pancreatic acinar cells^11,18–20^. In the present study, we confirmed that STIM1–Orai1 mediated the caerulein-induced SOCE and caused Ca^2+^ overload in pancreatic acinar cells. Consistent with previous studies^19,21^, we found that the severity of pancreatic injury, including pancreatic edema, inflammation and vacuole accumulation, was obviously attenuated by GSK7975A (CRAC channel blocker), which further demonstrate that STIM1–Orai1-mediated SOCE plays an influential role in AP (data not shown). However, the downstream mechanism of pancreatic injury triggered by SOCE need further investigation. Previous studies showed that both bile acid and CCK activated pancreatic CaN-NFAT signaling and resulted in pancreatic injury^41,42^. Our results showed that CaN activation depended on SOCE induced by caerulein. NFAT is an important downstream target of CaN, whose activation initiates a cascade of transcription of cytokine and immunoregulatory genes involved in physiologic and pathologic processes and results in inflammatory injury^49^. Therefore, our results imply that caerulein-induced SOCE activates CaN, which in turn initiates the transcription of cytokine genes and induces inflammatory cell infiltration and edema.

Vacuolization is one of the typical characteristics of pancreatic acinar cells in AP^37,50–52^. Previously, immunohistochemical and electron microscope studies suggested that the majority of vacuoles observed in AP were autophagic in origin. Meanwhile, previous studies suggested that autophagy was induced in pancreatitis^30–32^. Our data showed that the levels of total LC3, LC3II and SQSTM1/P62 were significantly increased and the transcription of LC3 and SQSTM1/P62 genes was promoted, indicating the inability of autophagosome turnover to keep pace with increased autophagosome formation. These results are consistent with previous reports^37,53^. We speculate that on the one hand more autophagosomes were formed with autophagy-associated proteins overexpressing, and on the other hand, because of the compromised fusion and lysosomal hydrolytic activity, autophagy flux is impaired and autophagosomes cannot be degraded in lysosome. This causes the autophagy-associated injury in AP, such as conversion of trypsinogen to trypsin, apoptosis and even necrosis^54^.

TFEB is a master regulator of lysosomal and autophagic function^33,55^. Phosphorylated TFEB is trapped in cytosol and remains inactive. When dephosphorylated, TFEB translocates to the nucleus and induces the transcription of target genes, such as LC3, SQSTM1/P62 and ATG9B^56,57^. Our experiment showed that the level of TFEB in pancreatitic mice was significantly increased and more TFEB was detected in the nuclei of pancreatic acinar cells that were treated with caerulein. These data indicate that TFEB may be a contributor of autophagy in AP. However, as described in autophagy guidelines^44^, the endogenous TFEB is difficult to detect and the cytoplasmic TFEB was also increased in pancreatic acinar cells after treatment with caerulein; we thus utilize confocal live cell imaging technique to investigate whether caerulein promotes the nuclear translocation of TFEB. We found that caerulein stimulation could induce TFEB to translocate from cytoplasm to nucleus in both Hela cells and pancreatic acinar cells expressing TFEB. TFEB is another downstream target of CaN, which have been confirmed recently^58^. Laboratory study showed that lysosomal Ca^2+^ signaling activates CaN, which in turn dephosphorylated TFEB and regulated its subcellular localization under starvation conditions^58^. We observed that both the inhibition of CaN and the block of CRAC channels restricted the nuclear translocation of exogenous and endogenous TFEB in Hela cell and pancreatic acinar cell respectively. These data suggest that SOCE-induced activation of CaN is a noticeable contributor to the nuclear translocation of TFEB in AP.

Furthermore, inhibition of SOCE or CaN reduced the formation of autophagosomes and the severity of vacuolization, edema and inflammation, which supports the hypothesis that CaN is the key regulator of autophagy and inflammation in AP.

In summary, our data indicated the link between SOCE, calcineurin and TFEB in pathogenesis of AP. We suggest caerulein-induced SOCE activated CaN, which in turn dephosphorylated TFEB and NFAT and respectively regulated autophagic and inflammatory injury in the development of AP. Considering that early onset of autophagy and vacuolization develop within 10–40 min from the beginning of the Ca^2+^ rise, this pathway is unlikely to have a pronounced effect on the fast process, but it may have longer-term effects on autophagy and vacuolization by regulating the expression and translocation of the autophagy-modifying genes in AP development. These data reveal that targeting CaN may be potential for arresting SOCE-induced pancreatic injury. In addition, it is tempting to speculate that, just as in AP, SOCE-regulating autophagy by CaN-mediated TFEB activation may also exist in some other SOCE-related diseases.

## Materials and methods

### Plasmid and virus

The plasmid NFAT1 (4–460)-GFP was a gift from Anjana Rao (Addgene plasmid # 11107). Full length of human TFEB and Orai1 were isolated by PCR, sequenced and cloned into pEGFP-N1 vector and mCherry-N1 vector respectively, and the resulting constructs were named TFEB-GFP and Orai1-mCherry. AAV-TFEB-EGFP was constructed by Vigene Bioscience Company. Fully synthesized mouse CCKAR complementary DNA (cDNA) was cloned into pECFP-N1 and mCherry-N1 vector, named CCKAR-CFP and CCKAR-mCherry respectively. STIM1-YFP encoding human full-length STIM1 is a reconstructed product based on the plasmid mCherry-STIM1 which was a gift from Dr Richard S (Stanford University, USA).

### Pancreatic acinar cell isolation

Pancreatic acinar cells were isolated from 25 to 30 g male C57BL/6 mice treated with type-4 collagenase digestion following a previously described method^59^ and were resuspended in Dulbecco’s modified Eagle's medium (DMEM)/F12 with 10% fetal bovine serum (FBS) or HEPES incubation buffer containing (in mM) 20 HEPES, 95 NaCl, 1.3 CaCl_2_, 4.7 KCl, 0.6 MgCl_2_, 10 glucose, 2 glutamine, 1× minimum essential medium (MEM) non-essential amino acids as well as 0.1% bovine serum albumin (BSA) and 0.01% soybean trypsinogen inhibitor (Ca^2+^-free solution had a similar composition but Ca^2+^ was omitted). All chemicals and reagents were from Sigma-Aldrich (USA) or Thermo Fisher Scientific (USA) except collagenase (Worthington, USA).

### Cell culture, transfection and live cell imaging

Hela cells and 293T cells (ATCC, USA) were grown in high-glucose DMEM (Gibco, USA) with 10% FBS (Gibco, USA). Cell cultures were incubated at 37 °C in 5% CO_2_ and 95% air humidified atmosphere. 293T cells and Hela cells were transfected using Lipofectamine 2000 reagent (Life Technologies, USA) according to the manufacturer’s instructions. To achieve more consistent results in the quantitative experiments of TFEB, Hela cells were transfected using electroporation. Briefly, Hela cells were trypsinized and washed 2 times with D-Hanks and 1 time with Opti-MEM (Gibco, USA) to remove BSA clearly, and then were resuspended in Opti-MEM to a concentration of 4.0 × 10^4^ cells/μL with 200 ng/μL plasmids. Then, 25 μL re-suspension was dropped in a gene pulser cuvette (Bio-Rad), and electric pulses (Pp V: 150 V, Pp on: 10 ms, Pp off: 10 ms, Pd V: 20 V, Pd on: 50 ms, Pd off: 50 ms, cycle: 10, capacitance: 940 μF) were applied with an electroporator (CUY21 EDIT II, BEX, Japan). After electroporation, cells were allowed to stand for about 30 min in gene pulser cuvette. Then, cells were distributed uniformly onto 30 mm round glass coverslips in 6-well cell culture plates. After 48 h, the glass coverslips were mounted in perfusion chamber and then time-lapse images of live cells were captured by a confocal microscope (Olympus Fluoview FV1000).

Pancreatic acinar cells were transfected with AAV-TFEB-EGFP for 24 h, and Hoechst 33258 (Beyotime, C1011) fluorescent staining was used to mark the nuclei 10 min before capturing by a confocal microscope.

FV10-ASW 3.0 viewer software (Olympus Optical Co., Tokyo, Japan) and Image-Pro Plus software (Media Cybernetics, Silver Spring, USA) were used for imaging data analysis.

### Calcium imaging

Coverslips (diameter = 30 mm) were mounted in perfusion chamber. Acutely dissociated acinar cells were plated on the coverslips and allowed to attach for 10 min, then were loaded with 2 μM high-affinity Ca^2+^-sensing dye fluo-4-AM (Invitrogen, USA) in HEPES incubation buffer for 30 min at room temperature, followed by washing and incubation for up to 30 min for intracellular deesterification of the dyes with 0.5 mL Ca^2+^-free HEPES incubation buffer.

Hela or 293T cells were transfected with CCKAR-mCherry. After 48 h, the transfected cells were plated onto 30 mm round glass coverslips and mounted in perfusion chamber. Cells were loaded with 2 μM fluo-4-AM for 20 min at 37 °C incubator in Ca^2+^ buffer containing (in mM) 1.8 CaCl_2_, 145 NaCl, 3 KCl, 2 MgCl_2_, 8 glucose and 10 HEPES, and then cells were washed and incubated for a further 20 min to de-esterify the dye with 0.5 mL Ca^2+^-free buffer containing (in mM) 145 NaCl, 3 KCl, 2 MgCl_2_, 8 glucose and 10 HEPES.

Fluorescence was recorded by time-series scan imaging on confocal microscope (Olympus Fluoview FV1000) at a wavelength of 488 nm. Single-image frames were acquired at an interval of 1 s using a 40× objective for a period of 10 min with the confocal aperture fully open. Data were analyzed by measuring emitted fluorescence from regions of interest over single cell using FV10-ASW imaging software. For Ca^2+^ add-back experiments, 10 nM caerulein (Bachem, Switzerland) or 2 μM thapsigargin (Sigma-Aldrich, USA) was used to induce Ca^2+^ store depletion, together with 5 mM EGTA to remove extracellular Ca^2+^. About 4 min later, 5 mM (in pancreatic acinar cells) or 2 mM (in Hela and 293T cells) Ca^2+^ was added back after Ca^2+^ store depletion. Where indicated, Orai1 inhibitor GSK7975A was added into the solution to a final concentration of 10 μM.

### Animal model of acute pancreatitis

C57BL/6 mice (weighing 25–30 g), supplied by the Experimental Animal Central of Tongji Medical College, were kept with ad libitum access to standard laboratory chow and water on a 12 h-light/dark cycle at 23 ˚C ± 2 °C. All animal experiments were approved by the Ethics Committee of Tongji Medical College. C57BL/6 mice were grouped randomly in all animal experiments. AP was induced via intraperitoneal injection of 50 µg/kg/h caerulein in 0.9% saline for 6 h, saline-injected animals served as controls, as previously described^60^. The pancreas was rapidly removed for further experiment 1 h after the final dosing. To determine the success of model establishing, serum amylase was measured using automatic biochemical analyzer in Clinic Laboratory of Wuhan Children’s Hospital and double-blinded histopathological analysis was performed as described before^61^ (data not shown).

### Sections and HE staining

The mice were fixed with 4% paraformaldehyde via transcardial perfusion, and tails of the pancreases were removed and immersed in 4% paraformaldehyde overnight at 4 ˚C before being embedded in paraffin. The pancreases were sectioned at a thickness of 5 µm. After drying, the sections were dewaxed in xylene and rehydrated step by step with descending concentrations of ethanol. Hematoxylin and eosin (HE) staining was performed to evaluate experimental acute pancreatic injury of pancreas including inflammation, edema and vacuolization under light microscope.

### RT-PCR analysis

Total RNA was extracted from pancreas using TRIzol reagent (Invitrogen, USA) according to the manufacturer’s instructions. RNA concentration and purity was measured spectrophotometrically at 260 and 280 nm. RNA was reverse-transcribed into cDNA using M-MLV reverse transcriptase (Promega, USA). Quantitative PCR amplification was performed using StepOnePlus system (ABI, USA) and SYBR Green Realtime PCR Master (TOYOBO, Japan) for LC3 and SQSTM1/P62. GAPDH was used as endogenous control. The relative quantity of target genes was calculated using the comparative cycle threshold method. The relative fold changes in mRNA expression level were calculated with the 2^−ΔΔCt^ method. The reaction conditions for real-time PCR is an initial denaturation at 95 °C for 1 min, followed by 40 cycles of 95 °C for 15 s, 60 °C for 15 s and extension at 72 °C for 45 s. The following primers were used:

**Table:** 

LC3 forward primer	5′-TTATAGAGCGATACAAGGGGGAG-3′
LC3 reverse primer	5′-CGCCGTCTGATTATCTTGATGAG-3′
SQSTM1/P62 forward primer	5′-GAACTCGCTATAAGTGCAGTGT-3′
SQSTM1/P62 reverse primer	5′-AGAGAAGCTATCAGAGAGGTGG-3′
GAPDH forward primer	5′-AGGTCGGTGTGAACGGATTTG-3′
GAPDH reverse primer	5′-GGGGTCGTTGATGGCAACA-3′

### Western blotting

The pancreatic tissue, acinar cells and Hela cells were homogenated and lysed in RIPA lysis buffer (ComWin Biotech, China) with 1 mM phenylmethylsulfonyl fluoride and 0.1% Cocktail for 30 min on ice. Then, centrifugation at 12,000 rpm for 15 min at 4 °C and the supernatant was collected. The nuclear fraction was prepared by using the Nuclear and Cytoplasmic Extraction kit (ComWin Biotech, China) following the manufacturer’s instructions. The samples were boiled at 95 °C in the loading buffer, and then were separated by 10–15% SDS–polyacrylamide gel electrophoresis and transferred onto 0.45 µm nitrocellulose blotting membranes (GE Healthcare, USA).The membranes were blocked in 5% skim milk dissolved in phosphate-buffered saline (PBS) for 30 min and incubated with primary antibodies TFEB (1:500, rabbit polyclonal, Santa Cruz, sc-48784x), LC3 (1:500, rabbit polyclonal, Protein Tech, 12135-1-AP), STIM1 (1:500, rabbit polyclonal, Sigma-Aldrich, S6197), Orai1 (1:500, rabbit polyclonal, Protein Tech, 13130-1-AP), β-actin (1:2000, mouse monoclonal, Protein Tech, 66009-1-Ig), GAPDH (1:2000, mouse monoclonal, Protein Tech, 60004-1-Ig) overnight. Following 3 times washing with PBS for 10 min each, the membranes were incubated with IRDye 800-labeled secondary antibody (1:10,000, Goat Anti-Rabbit or Donkey Anti-Mouse, Li-Cor Biosciences, 926-32211 or 926-32212) for 2 h at room temperature, then washed 3 times with PBS, visualized and quantified using the Odyssey Infrared Imaging System (Li-Cor Biosciences).

### CaN activity assay

Pancreatic acinar cells were acutely isolated and were pretreated with dimethyl sulfoxide (DMSO) or 10 µM FK506 or 10 µM GSK7975A or 5 mM EGTA for 30 min before stimulating with 10 nM caerulein for 15 min. The cells were lysed and the activity of CaN was assayed by using a calcineurin cellular activity assay kit (Abcam, ab139464) by following the manufacturer’s instructions.

### Statistical analysis

Statistical analyses were carried out using SPSS statistics 19.0 (SPSS Inc., Chicago, USA). All the data were presented as mean ± SEM from at least three experiments. Statistical significance was tested using either Student’s *t*-test or one-way analysis of variance. The test with *p* < 0.05 was considered as statistically significant.
